# Supplementary material for: Gentiopicroside-Induced gastric cancer necroptosis via the HIF-1 signaling pathway: A study involving molecular docking and experimental validation
Source: PLoS One. 2024 Nov 21;19(11):e0311152. doi: 10.1371/journal.pone.0311152 (PMC11581292; doi:10.1371/journal.pone.0311152)
Supplement: S1 Raw images — (PDF) [file pone.0311152.s002.pdf]

Fig 2D. HIF-1α

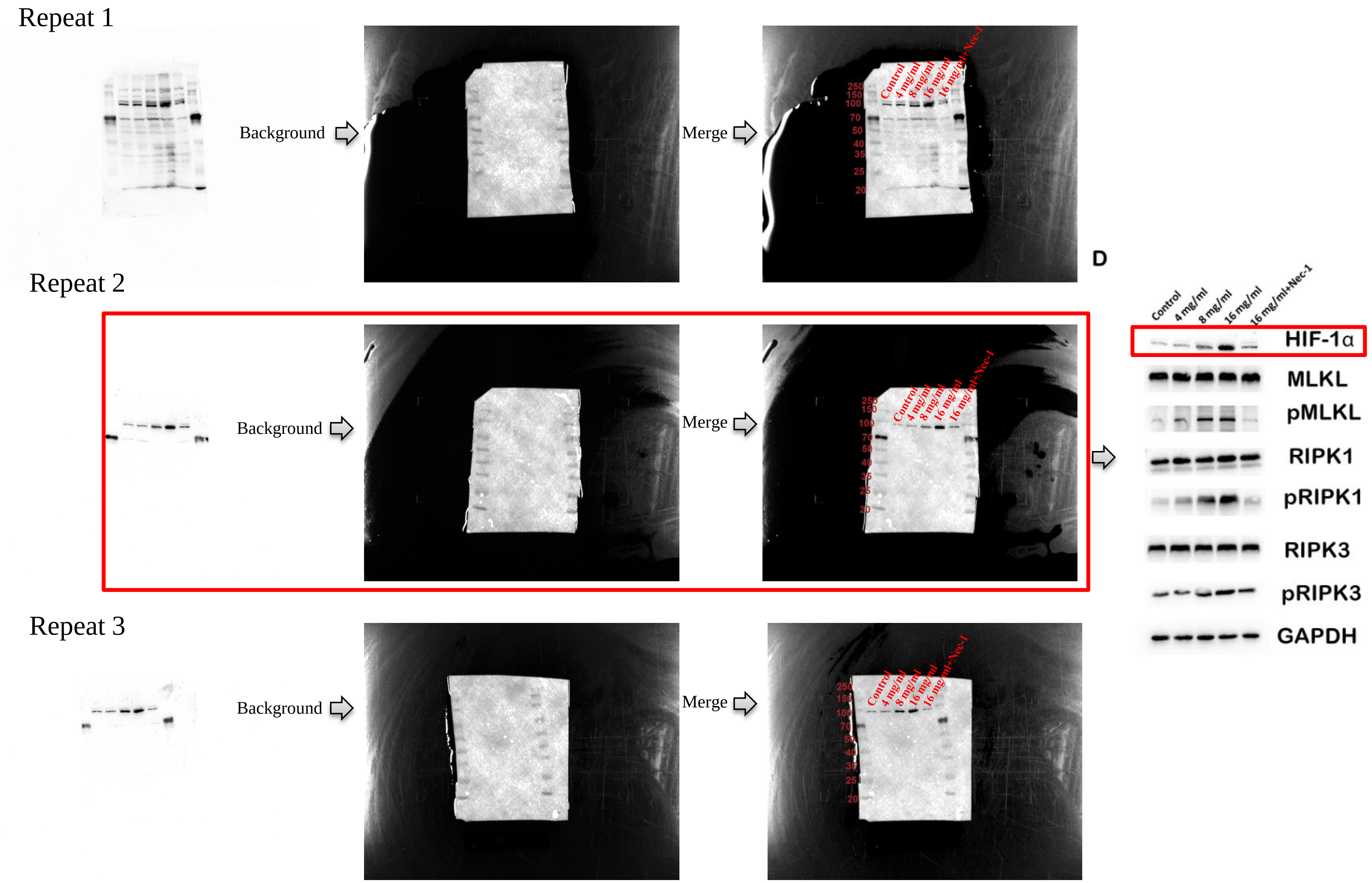

Repeat 1
Background
Merge
250
150
100
70
50
40
35
25
20
Control
4 mg/ml
8 mg/ml
16 mg/ml
16 mg/ml+Nec-1
Repeat 2
Background
Merge
Repeat 3
Background
Merge
D
HIF-1α
MLKL
pMLKL
RIPK1
pRIPK1
RIPK3
pRIPK3
GAPDH

Fig 2D. MLKL

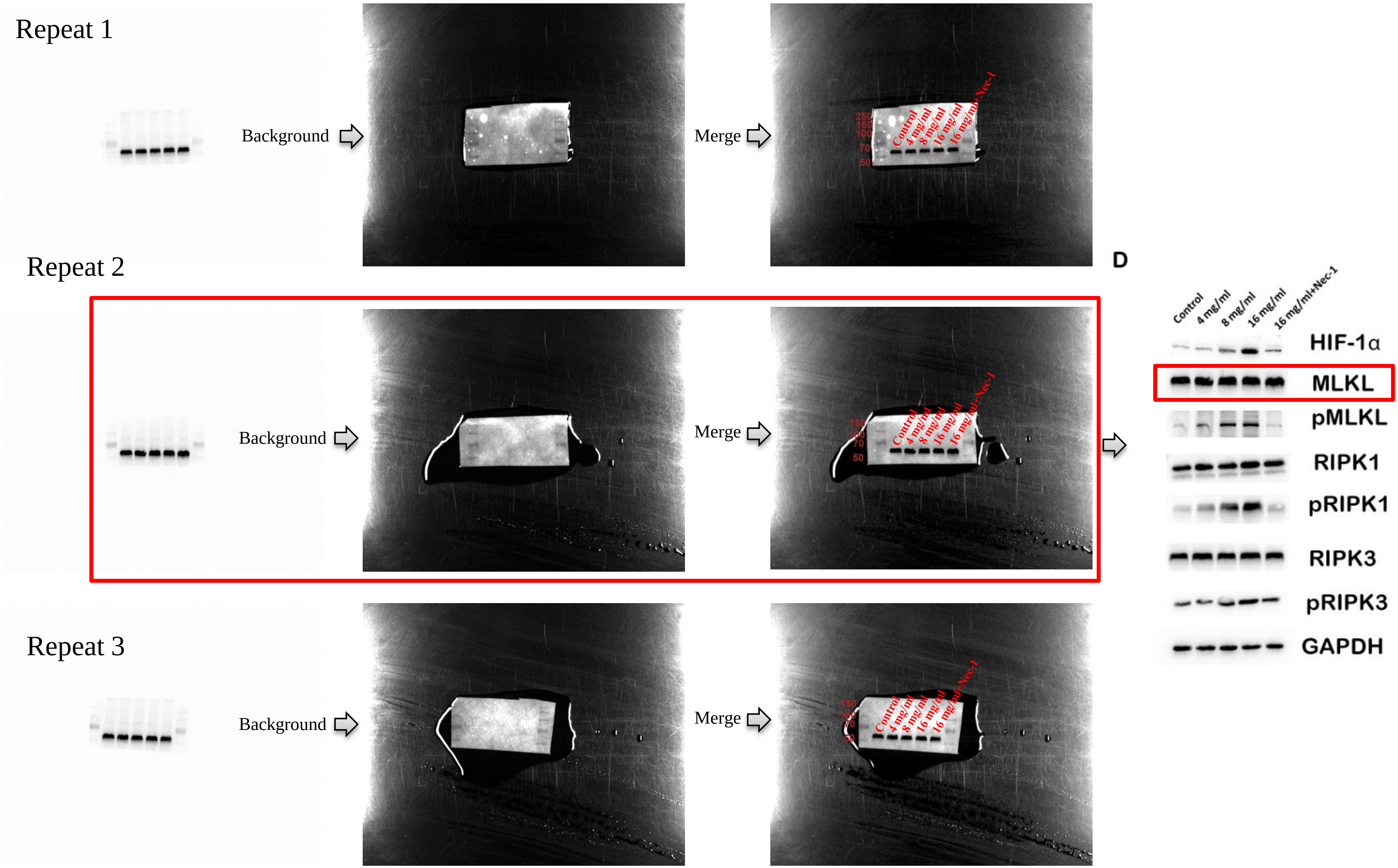

Repeat 1
Background
Merge
Repeat 2
Background
Merge
Repeat 3
Background
Merge
D
Control
4 mg/ml
8 mg/ml
16 mg/ml
16 mg/ml+Nec-1
HIF-1α
MLKL
pMLKL
RIPK1
pRIPK1
RIPK3
pRIPK3
GAPDH

Fig 2D. pMLKL

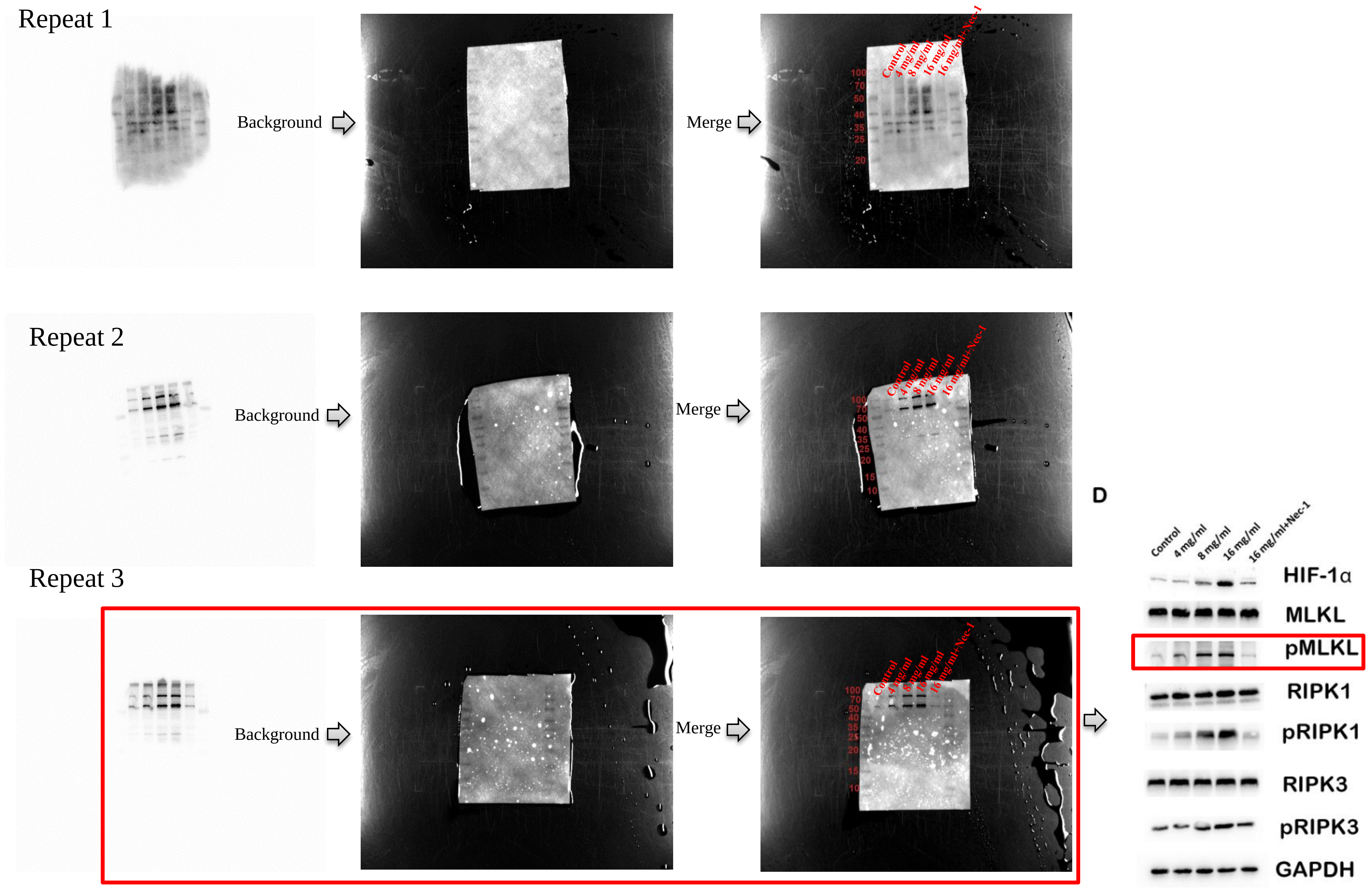
Repeat 1
Background
Merge
Control
4 mg/ml
8 mg/ml
16 mg/ml
16 mg/ml+Nec-1
100
70
50
40
35
25
20
Repeat 2
Background
Merge
Control
4 mg/ml
8 mg/ml
16 mg/ml
16 mg/ml+Nec-1
100
70
50
40
35
25
20
15
10
Repeat 3
Background
Merge
Control
4 mg/ml
8 mg/ml
16 mg/ml
16 mg/ml+Nec-1
100
70
50
40
35
25
20
15
10
D
Control
4 mg/ml
8 mg/ml
16 mg/ml
16 mg/ml+Nec-1
HIF-1α
MLKL
pMLKL
RIPK1
pRIPK1
RIPK3
pRIPK3
GAPDH

Fig 2D. RIPK1

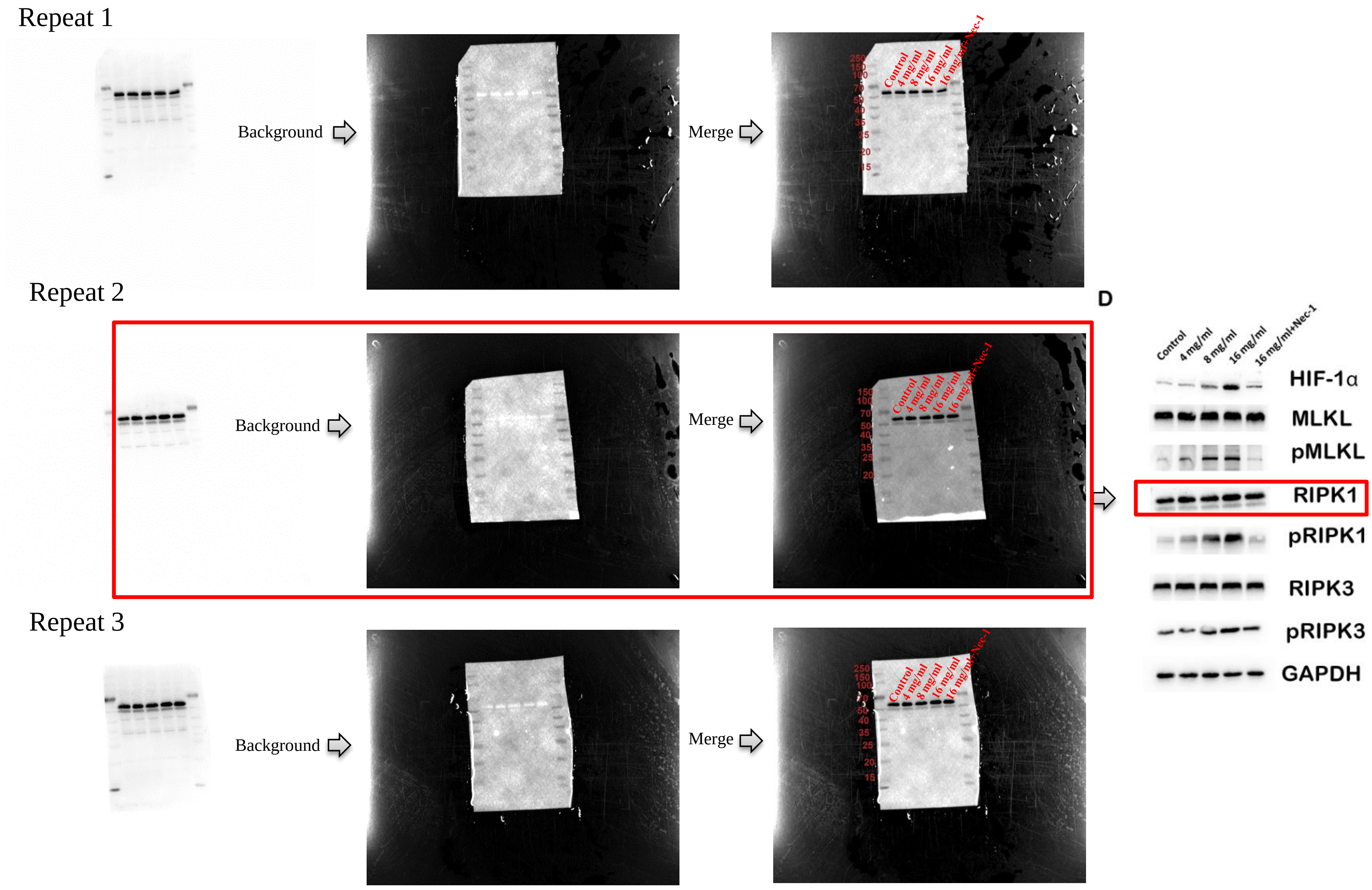
Repeat 1
Background
Merge
250
150
100
70
50
40
35
25
20
15
Control
4 mg/ml
8 mg/ml
16 mg/ml
16 mg/ml+Nec-1
Repeat 2
Repeat 3
D
HIF-1α
MLKL
pMLKL
RIPK1
pRIPK1
RIPK3
pRIPK3
GAPDH

Fig 2D. pRIPK1

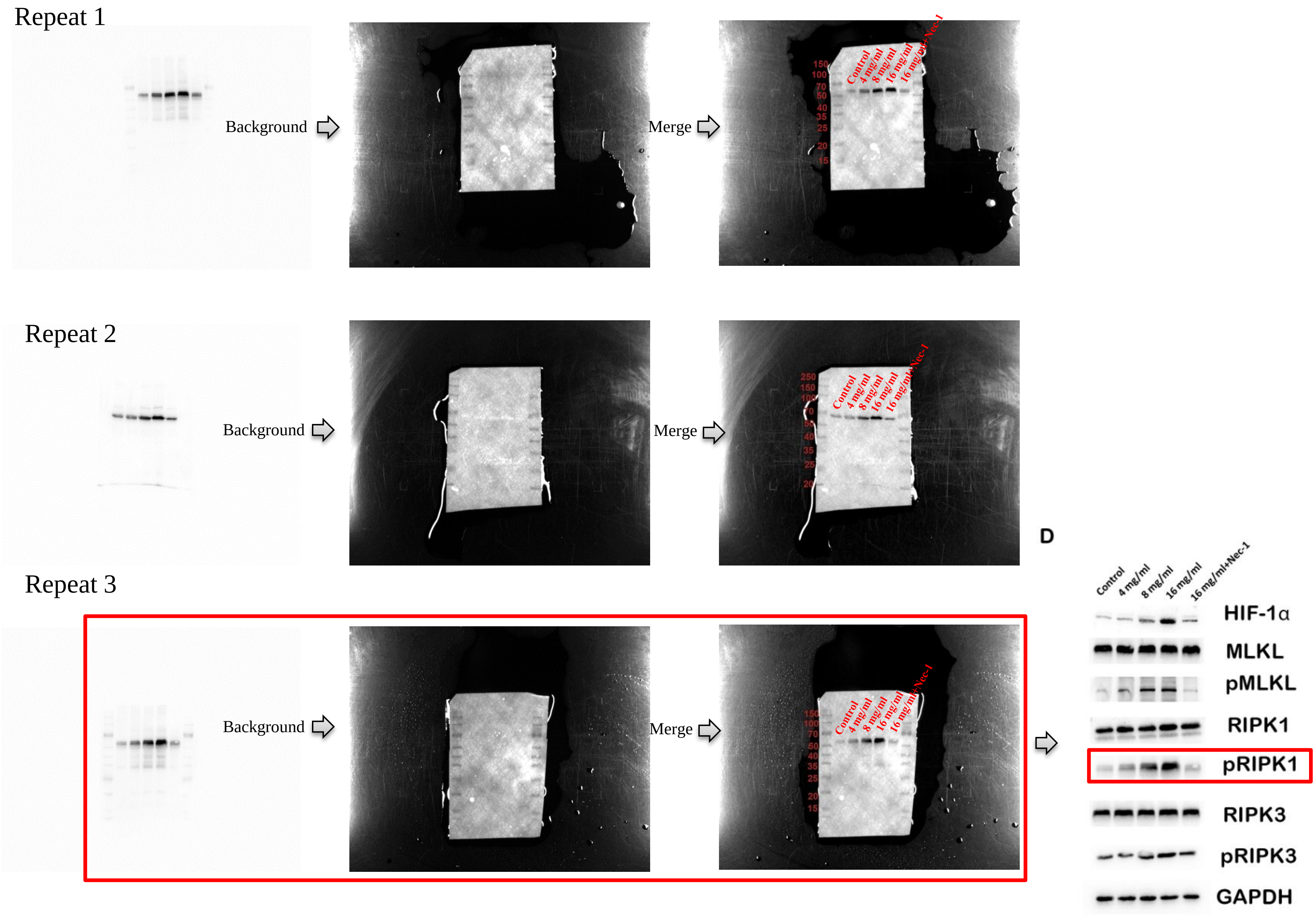

Fig 2D. RIPK3

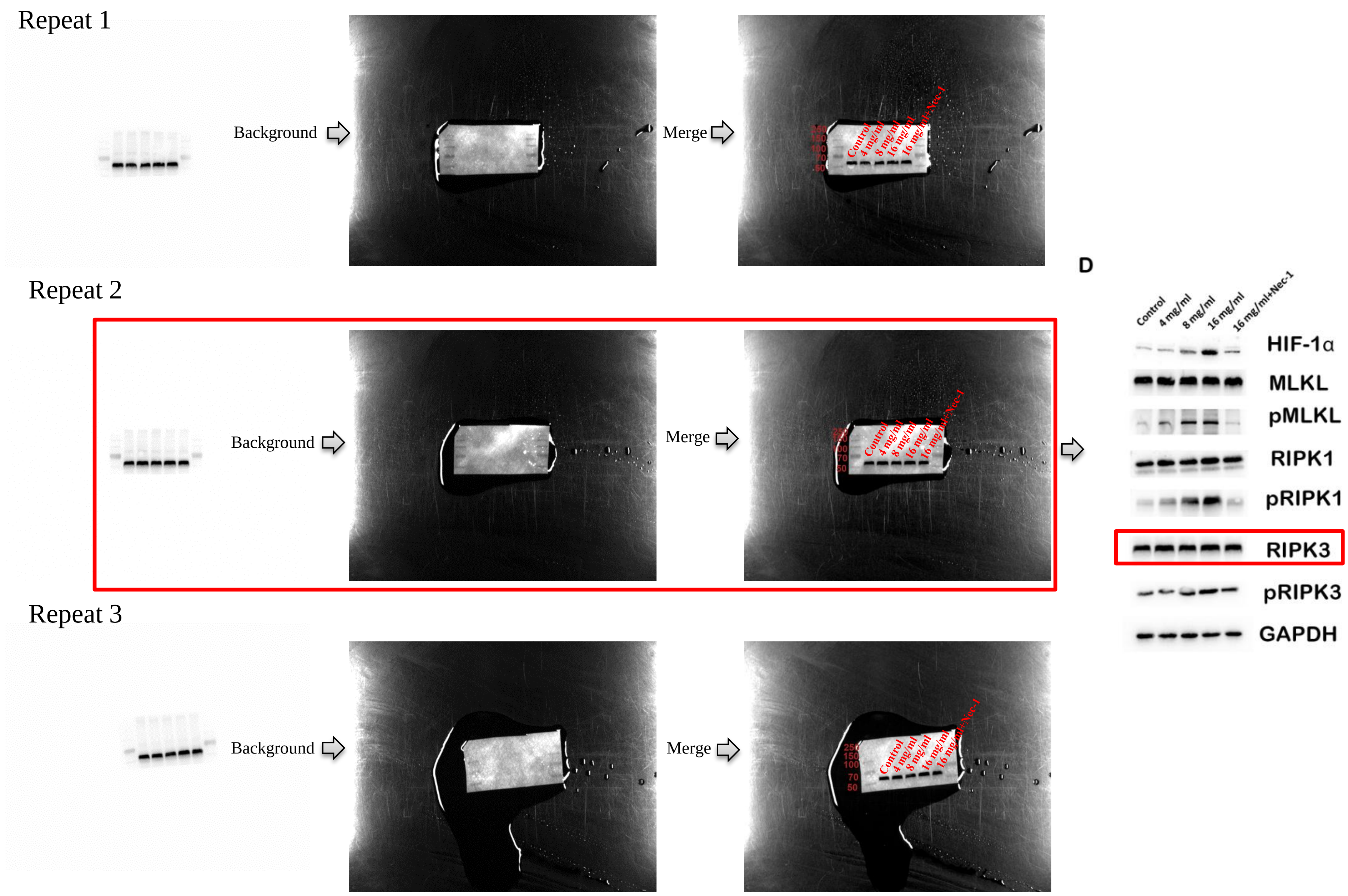

Repeat 1
Background
Merge
Repeat 2
Background
Merge
Repeat 3
Background
Merge
D
Control
4 mg/ml
8 mg/ml
16 mg/ml
16 mg/ml+Nec-1
HIF-1α
MLKL
pMLKL
RIPK1
pRIPK1
RIPK3
pRIPK3
GAPDH

Fig 2D. pRIPK3

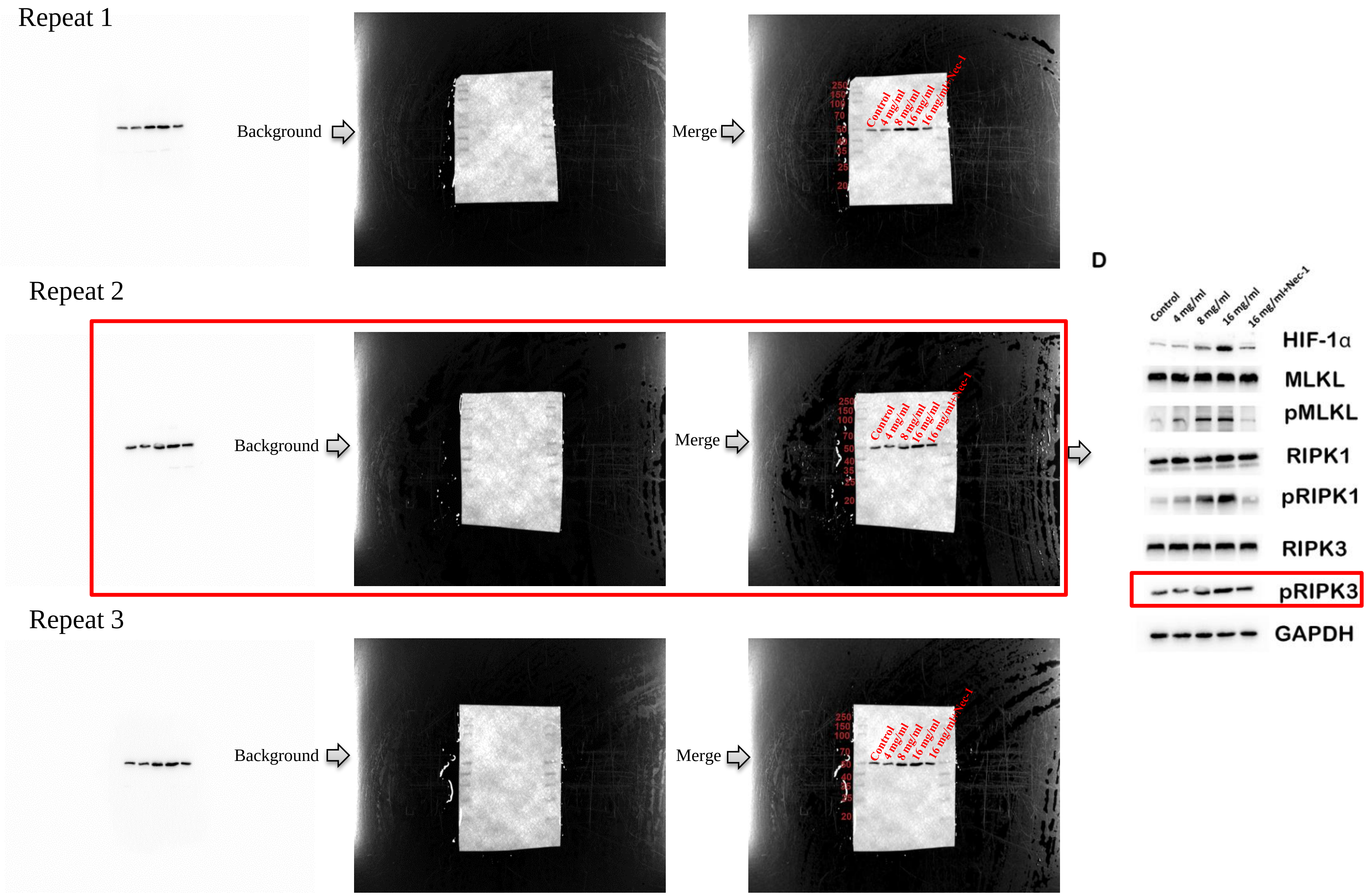

Fig 2D. GAPDH

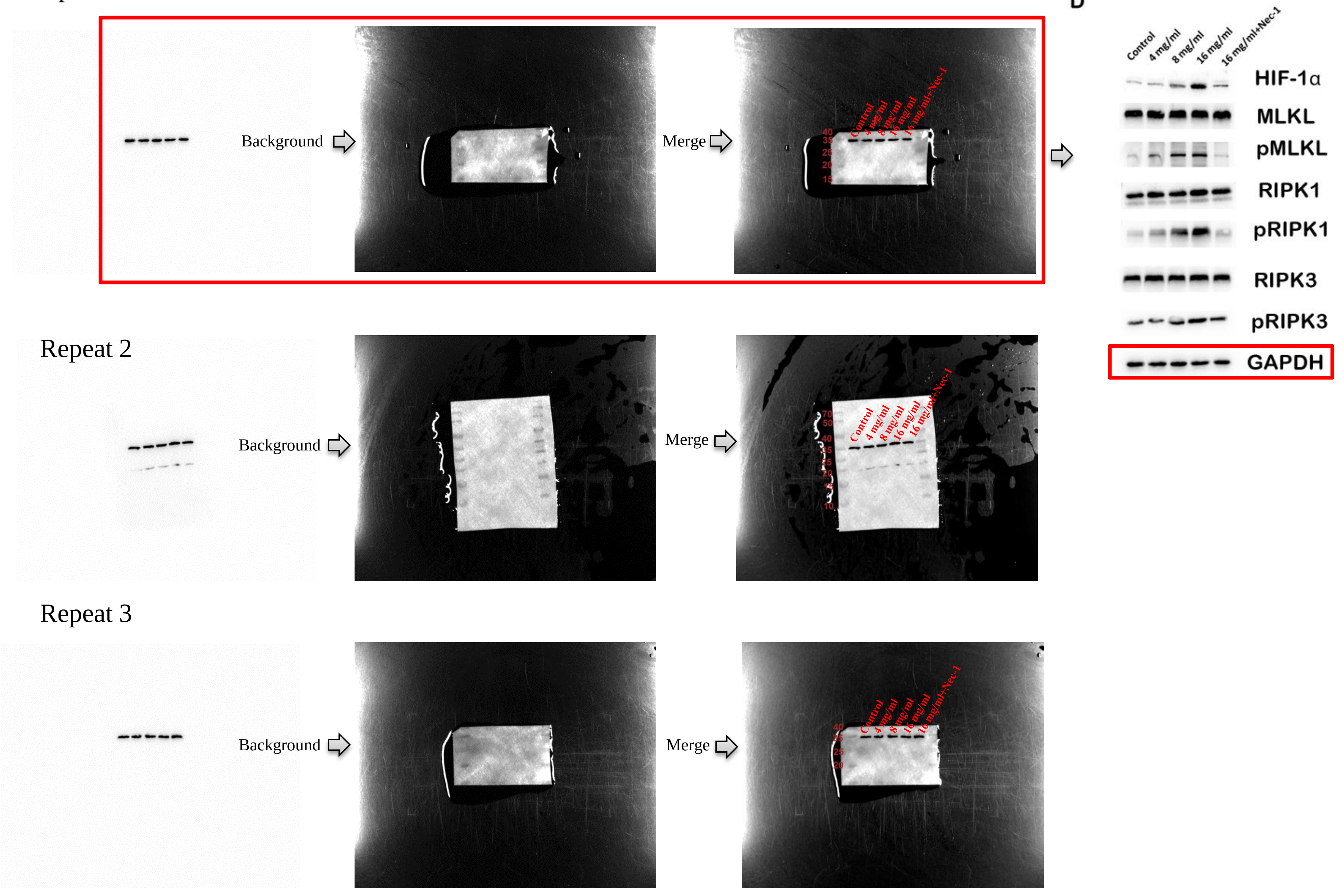

Repeat 1
Background
Merge
Repeat 2
Background
Merge
Repeat 3
Background
Merge
Control
4 mg/ml
8 mg/ml
16 mg/ml
16 mg/ml+Nec-1
D
Control
4 mg/ml
8 mg/ml
16 mg/ml
16 mg/ml+Nec-1
HIF-1α
MLKL
pMLKL
RIPK1
pRIPK1
RIPK3
pRIPK3
GAPDH
